# Development of an instrument to measure medical students’ perceptions of the assessment environment: initial validation

**DOI:** 10.3402/meo.v20.28612

**Published:** 2015-10-27

**Authors:** Joong Hiong Sim, Wen Ting Tong, Wei-Han Hong, Jamuna Vadivelu, Hamimah Hassan

**Affiliations:** Faculty of Medicine, University of Malaya, Kuala Lumpur, Malaysia

**Keywords:** development, instrument, assessment environment, psychometric properties, validation

## Abstract

**Introduction:**

Assessment environment, synonymous with climate or atmosphere, is multifaceted. Although there are valid and reliable instruments for measuring the educational environment, there is no validated instrument for measuring the assessment environment in medical programs. This study aimed to develop an instrument for measuring students’ perceptions of the assessment environment in an undergraduate medical program and to examine the psychometric properties of the new instrument.

**Method:**

The Assessment Environment Questionnaire (AEQ), a 40-item, four-point (1=Strongly Disagree to 4=Strongly Agree) Likert scale instrument designed by the authors, was administered to medical undergraduates from the authors’ institution. The response rate was 626/794 (78.84%). To establish construct validity, exploratory factor analysis (EFA) with principal component analysis and varimax rotation was conducted. To examine the internal consistency reliability of the instrument, Cronbach's *α* was computed. Mean scores for the entire AEQ and for each factor/subscale were calculated. Mean AEQ scores of students from different academic years and sex were examined.

**Results:**

Six hundred and eleven completed questionnaires were analysed. EFA extracted four factors: feedback mechanism (seven items), learning and performance (five items), information on assessment (five items), and assessment system/procedure (three items), which together explained 56.72% of the variance. Based on the four extracted factors/subscales, the AEQ was reduced to 20 items. Cronbach's *α* for the 20-item AEQ was 0.89, whereas Cronbach's *α* for the four factors/subscales ranged from 0.71 to 0.87. Mean score for the AEQ was 2.68/4.00. The factor/subscale of ‘feedback mechanism’ recorded the lowest mean (2.39/4.00), whereas the factor/subscale of ‘assessment system/procedure’ scored the highest mean (2.92/4.00). Significant differences were found among the AEQ scores of students from different academic years.

**Conclusions:**

The AEQ is a valid and reliable instrument. Initial validation supports its use to measure students’ perceptions of the assessment environment in an undergraduate medical program.

Literature in medical education is abundant in studies exploring or assessing students’ perceptions of the learning or educational environment in medical programs in general (1–7), in certain disciplines, or training programs such as chiropraxy, veterinary medicine, and physiotherapy (8–10), as well as in specific settings such as in a distributed medical program (11) and clinical rotations in undergraduate medical programs (12, 13).

Although assessment is an integral part of the teaching and learning process and plays a central role in any form of education, research that investigates medical students’ perceptions of the assessment environment is scarce. Apart from studies on students’ perceptions of assessment and feedback in longitudinal integrated clerkships (14), redesigning learning and assessment environment (15), and two older studies on students’ views on continuous assessment at the University of Birmingham (16, 17), research related to assessment environment is not as robust compared to that of learning or educational environment.

Medical schools face the challenges of designing and implementing assessment programs that are effective, practical, acceptable, and defensible to all the stakeholders in the healthcare services – medical students, medical teachers, medical schools, and the society. Scrutiny of the assessment system and related matters from time to time could reassure all stakeholders that it continues to achieve its goals. Equally important is to ensure the assessment environment is favourable for students’ learning and performance.

Assessment environment, synonymous with climate or atmosphere, is multifaceted. It encompasses a multitude of diverse factors associated with assessment practices such as the assessment system/procedure, information on assessment, feedback mechanism, and assessment methods and/tools. Medical students are the ones directly affected by the assessment environment. The assessment system, assessment procedure, selection of assessment methods and/tools, as well as feedback mechanism not only affect their learning processes but also their performance. As stakeholders, students’ perceptions of the assessment environment are important and their feedback deserves attention.

To date, there is no documented study or any form of evidence-based data on students’ perceptions of the assessment environment of the medical program in the University of Malaya (UM). A study on medical students’ perceptions of the assessment environment of the medical program in UM is timely and appropriate.

There are several valid and reliable instruments for measuring the educational environment such as the Dundee Ready Education Environment Measure, the Postgraduate Hospital Educational Environment Measure, and numerous studies which attempt to develop and validate instruments for measuring the educational environment (18–24). In contrast, there is no valid and reliable instrument for measuring the assessment environment in medical programs. Although there is an instrument – the Perceived Classroom Environment Scale developed by Alkharusi (25), this instrument was used to measure high school students’ perceptions of the classroom assessment environment (26) and might not apply to undergraduates in a medical program.

Therefore, this study aimed to develop and test an instrument that could be used to measure the assessment environment in an undergraduate medical program. Specifically, the objectives of the study were: 1) to develop an instrument to measure students’ perceptions of the assessment environment in the medical program in the UM, and 2) to examine the psychometric properties (face, content, and construct validity, and internal consistency reliability) of the new instrument. In the context of this study, students’ perception of the assessment environment refers to the overall sense or meaning that medical students make out of the assessment practices in the undergraduate medical program.

## Method

### Construction of items in the questionnaire

Based on available literature (16, 17), observation of the assessment environment of the undergraduate medical program in the authors’ institution, as well as informal interviews with faculty members and medical students from different academic years, several factors that appeared to have influence on the assessment environment were noted. These included assessment system/procedure, assessment methods and/tools, information on assessment, and feedback. Following that, potential items related to these factors were written and randomly placed to generate the draft questionnaire.

To check for face and content validity, the draft questionnaire with 41 items was reviewed by a panel comprising two academic staff, one research officer, one laboratory staff, and one PhD student in medical education. All members of the panel were from the Medical Education and Research Development Unit, Faculty of Medicine, in the authors’ institution. They agreed that the draft questionnaire was initially face and content valid.

### Pilot study

Pilot study began after ethics clearance was granted by the medical ethics committee (MEC) of University of Malaya Medical Centre on 30 October 2013 (MEC Ref. No: 1024.77). The 41-item draft questionnaire was pilot tested with 39 final year medical students from the authors’ institution. Cronbach's *α* of 0.94 was recorded across the 41 items. A discussion session was subsequently held where two researchers of this study asked the 39 respondents for comments on the questionnaire. Issues such as ambiguity and clarity of the statements in the draft questionnaire were discussed. Based on the feedback, one item (Item 15) with the statement ‘Student's clinical performance is appropriately assessed during ward rounds and case presentations’, which was viewed as redundant with the statement ‘Students’ clinical skills are appropriately assessed using OSCE, long case and short case’ (Item 39), was deleted from the draft questionnaire. Following the deletion, the item number of the statements also changed accordingly. For example, Item 16 was renumbered as Item 15, and so on. Several items were also rephrased or reconstructed. For example, the statement ‘Feedback is given within a short time (≈3 weeks) after an assessment’ had been rephrased as ‘Feedback is given promptly after an assessment’, and another statement ‘The feedback I received is effective in improving my learning’ had been reconstructed as ‘The feedback I received helped me to improve my learning’.

### Study instrument

The instrument for the actual study, the Assessment Environment Questionnaire (AEQ) is a 40-item questionnaire in a four-point Likert scale ranging from 1 (Strongly Disagree) to 4 (Strongly Agree). Items that were worded negatively (Item 11 and Item 13) were reverse-scored, with a score of 1 for ‘Strongly Agree’ and a score of 4 for ‘Strongly Disagree’. Total possible score for the 40-item AEQ should be 4×40=160. However, for the 20-item AEQ after the final analysis, total possible score for the entire AEQ was 4×20=80. A copy of the 40-item questionnaire is provided in Appendix 1.

### Overview of institutional setting/study context

The University of Malaya Bachelor of Medicine and Bachelor of Surgery (MBBS) degree is a 5-year program. The program is divided into three phases: Phase 1 (1 year), Phase 2 (1 year), and Phase 3 (3 years). Phase 3 (clinical years) is further divided into Phase 3A and Phase 3B of 1.5 years each. Currently, there are two medical curricula coexisting: the New Integrated Curriculum (NIC) and the University of Malaya Medical Programme (UMMP). At the time of data collection, only Phase 1 students were following the UMMP. Students from Phase 2, Phase 3A, and Phase 3B followed the NIC. Although the NIC is more discipline-based with basic sciences for the first 2 years and clinical teaching usually begins in the third year, the UMMP is multidisciplinary with early clinical exposure beginning in the first year. In terms of assessment, the three main assessment formats (written, practical, and clinical) are used for both curricula. However, there are two main differences. Firstly, in written examinations, the question type of true/false is used in the NIC, whereas single best answer and extended matching question types are used in the UMMP. Secondly, for the NIC, clinical assessments only begin in clinical years, whereas the UMMP clinical assessments begin in the first year.

### Study sampling

The sampling design for this study was cross-sectional. Universal systematic sampling was adopted to cover all the medical students in the UM.

### The study population

The total enrolment of medical undergraduates in our institution was 794 (Phase 1=179, Phase 2=204, Phase 3A=208, and Phase 3B=203). In total 626 students (78.84%) responded to the questionnaire. In terms of academic years, Phase 1=166, Phase 2=181, Phase 3A=162, and Phase 3B=117. In terms of sex, there were 231 males and 395 females. The respondents from the pilot testing (*n=*39) were not included in the actual survey. Out of the 626 questionnaires received, 15 questionnaires were removed because of incomplete responses and excluded from analysis.

### Data collection

The survey was conducted from November 2013 to June 2014. Researchers explained to the students the objectives of the study prior to distributing the questionnaire for self-administration among the respondents after their lecture sessions, in four separate sessions, according to the academic year of the respondents.

### Statistical analysis

Analyses of items in the questionnaire were conducted using IBM SPSS Statistics version 22. Both descriptive and inferential statistics were used.

To check on the construct validity of the instrument, the factor structure of the AEQ was determined through exploratory factor analysis (EFA) with principal component analysis (PCA) and varimax rotation. Factor analysis is a technique for identifying groups or clusters of variables that relate to each other (27, 28). In this study, EFA was used as the researchers/authors wanted to explore the main dimensions related to the assessment environment as represented by a set of variables or items that describe the assessment environment, and to summarise the structure of the set of variables or items (28). PCA with varimax rotation was opted as the researchers/authors believed there were no grounds for supporting that the variables might correlate (28).

To examine the internal consistency reliability of the instrument and its subscales, Cronbach's *α* was computed across all the items as well as for items within each of the factor/subscales for all the completed questionnaires (*n=*611). Mean total score and standard deviation for the entire AEQ as well as mean score for each of the subscales were computed using descriptive statistics.

One-way ANOVA was used to compare mean total score of the entire AEQ for different academic years, whereas independent sample *t*-tests were used to compare mean total score of the entire AEQ for male and female students. An alpha level of 0.05 was set for all the statistical tests.

## Results

A summary showing the enrolment of medical undergraduates, number of respondents, and response rate for the AEQ is provided (Table 1).

**Table 1:** Response rate for the survey questionnaires

Academic year	Enrolment	No. of respondents	Complete QA	% response
Phase 1	179	166	161	92.74
Phase 2	204	181	178	88.73
Phase 3A	208	162	156	79.80
Phase 3B	203	117	116	57.64
Total	794	626	611	78.84

In preliminary analysis, EFA with PCA and varimax rotation yielded six factors with eigenvalues over Kaiser's criterion of 1 (29). The six factors together accounted for approximately 47% of the variance. Seven items (Items 5, 12, 14, 22, 24, 31, and 33) with commonalities below 0.40 were removed. Another eight items (Items 3, 10, 20, 21, 23, 32, 37, and 38) with cross-factor loadings were discarded. All these eight items had factor loadings less than 0.50 but loaded onto two factors, each with a factor loading of more than 0.30 (Appendix 2). Out of these 15 deleted items, all except Item 5 had factor loadings less than 0.50 (Appendix 2). The 40-item AEQ was subsequently reduced to 25 items.

Cronbach's *α* for the 25-item AEQ was 0.86, whereas Cronbach's *α* for the six factors/subscales ranged from 0.44 to 0.87.

Although the factor loadings for Item 11 and Item 13 in factor F6 were 0.538 and 0.728 respectively, an attempt was made to exclude F6 because this factor/subscale contained only two items and did not have good internal consistency (*α=*0.44). With Items 11 and 13 deleted, Cronbach's *α* for the 23-item AEQ with five factors/subscales increased to 0.89.

Another factor F5 has only moderate internal consistency (*α=*0.52) with three items (Items 26, 30, and 39). Because factor analysis provides statistical guidelines to a researcher in determining factor structure of an instrument and should not be viewed as too rigid a procedure to follow strictly (28), attempts were made to include these three items under the factors containing variables or items of apparently related content. Therefore, Item 30 was placed in F4 and Items 26 and 39 were placed in F3. However, the inclusion of these items in the said factors/subscales led to F4, F3, and the overall AEQ recorded lower *α* values. Hence, a decision was made to exclude F5, resulting in a 20-item AEQ with four factors/subscales. Cronbach's *α* for the 20-item AEQ remained at 0.89.

The Kaiser–Meyer–Olkin (KMO) and Barlett's test of sphericity produce the KMO measure of sampling adequacy and Barlett's test (28). A value of KMO close to 1 indicates that pattern of correlations are relatively compact and so factor analysis should yield distinct and reliable factors. Kaiser (29) recommends KMO values of greater than 0.5 acceptable. In addition, according to Hutcheson and Sofroniou (30), values between 0.5 and 0.7 are mediocre, values between 0.7 and 0.8 are good, and values between 0.8 and 0.9 are great. The Barlett's test must be significant for factor analysis to work (28). In the final analysis, the KMO measure with KMO=0.90 which is well above the acceptable limit of 0.50, verified the sampling adequacy for factor analysis (28). The Bartlett's test of sphericity is highly significant, with χ^2^ (190)=4442.97, *p*<0.001, indicating that correlations between items were sufficiently large for factor analysis to be conducted (28). All extraction communalities ranged from 0.43 to 0.72, indicating the sample size (*n=*611) was adequate for factor analysis (28).

**Fig. 1:**
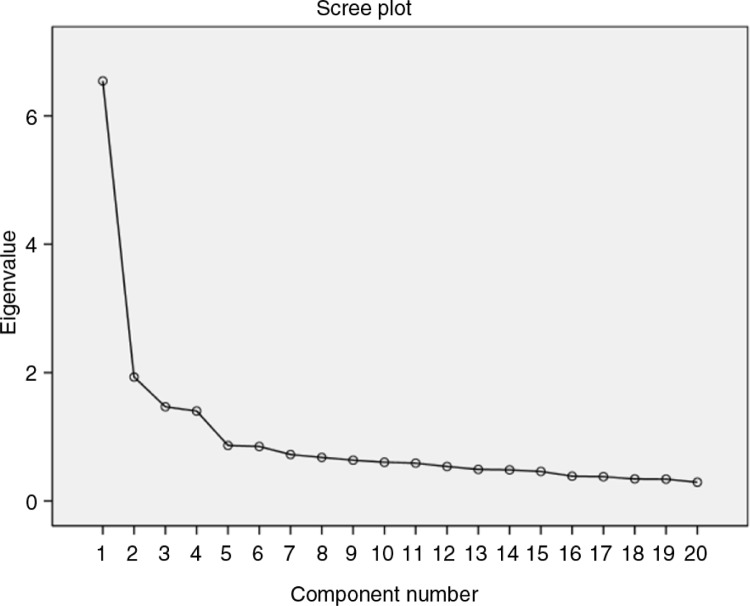
The scree plot.

Four factors with eigenvalues of 6.54, 1.93, 1.47, and 1.40, which together explained 56.72% of the variance, were retained. The scree plot is a useful way of establishing how many factors should be retained in an analysis (28). Examination of the scree plot (Fig. 1), which showed the line began to flatten at component number 5, justified the retention of these four factors.

Compared to the six-factor AEQ in the preliminary analysis which only accounted for approximately 47% of the variance, the 4-factor AEQ explained 56.72% of the variance.

A decision was made to adopt the 20-item, 4-factor AEQ for the purpose of validating the new instrument. Table 2 provides a summary of EFA results for the 20-item AEQ (*n=*611).

**Table 2:** Summary of exploratory factor analysis results for the 20-item AEQ (*n=*611)

		Rotated factor loadings			
		
Item no.	Statement	F1	F2	F3	F4
8	I received feedback on my performance for continuous assessment.	0.58			
17	I received feedback on my performance for final exams.	0.76			
18	Feedback from assessors about my performance is adequate.	0.77			
19	Feedback is given promptly after an assessment.	0.82			
27	The form of feedback I received matches the purposes of the assessments.	0.58			
28	Feedback from assessors about my performance is appropriate.	0.64			
29	I receive on-going feedback on my progress.	0.71			
9	The assessment system encourages me to reflect on my own performance.		0.51		
34	I receive feedback on my work from a range of sources (e.g., teachers, peers)		0.53		
35	The feedback I received helped me to improve my learning.		0.83		
36	The assessment system supports my learning.		0.61		
40	The feedback I received helped me to improve my grades.		0.74		
6	A description of how individual assessments and exams contribute to the total score is made known to students.			0.61	
7	I received information about what is expected of me in any exam/assessment.			0.64	
15	Students receive clear information about assessment.			0.76	
16	I understand the assessment criteria for all the tests/exams I took.			0.75	
25	Assessment criteria are clearly defined.			0.58	
1	Assessment in the MBBS programme is conducted fairly.				0.82
2	Students are adequately assessed.				0.79
4	Learning outcomes are appropriately assessed.				0.71
Eigenvalues		6.54	1.93	1.47	1.40
% of variance		19.34	14.12	13.19	10.07
Total variance					57.62

Note: F1=feedback mechanism, F2=learning and performance, F3=information on assessment, F4=assessment system/procedure.

Reliability analysis reported an overall Cronbach's *α* of 0.89 for the 20-item AEQ. Cronbach's *α* for the four subscales ranged from 0.71 to 0.87 (Table 3).

**Table 3:** Internal consistency of the AEQ

Factor/subscale	No. of items	Item no.	Cronbach's alpha coefficient
Feedback mechanism	7	8, 17, 18, 19, 27, 28, 29	0.87
Learning and performance	5	9, 34, 35, 36, 40	0.76
Information on assessment	5	6, 7, 15, 16, 25	0.75
Assessment system/procedure	3	1, 2, 4	0.71
Overall AEQ	20		0.89

### Mean score

Mean total score for the 20-item AEQ was 53.63±6.83/80.00 (or 67.04%), whereas mean score for the individual AEQ items was 2.68/4.00 (67.05%).

Mean score for the four subscales of the AEQ were: 2.39/4.00 (feedback mechanism), 2.84/4.00 (learning and performance), 2.74/4.00 (information on assessment), and 2.92/4.00 (assessment system/procedure).

### Comparing means

Mean scores for the AEQ by academic year were 2.68±0.28, 2.81±0.26, 2.67±0.33, and 2.61±0.30 for Phase 1, Phase 2, Phase 3A, and Phase 3B respectively. One-way ANOVA found significant differences among the AEQ scores from students of different academic years [*F* (3, 608)=9.918, *p*<0.001]. *Post hoc* Scheffe test further revealed significant differences between the following three pairs of means, at *p*<0.001. These were: Phase 2 versus Phase 1, Phase 2 versus Phase 3A, and Phase 2 versus Phase 3B.

Mean scores for the AEQ by sex were 2.68±0.34 and 2.72±0.27 for males and females respectively. However, independent sample *t*-tests showed no significant difference between the AEQ scores of male and female students, at *p*<0.05.

## Discussion

After examining the content of the items that loaded onto the same factor to try to identify common themes, these four factors were labelled as: F1 – feedback mechanism (seven items), F2 – learning and performance (five items), F3 – information on assessment (five items), and F4 – assessment system/procedure (three items). These four factors together explained 56.72% of the variance (Table 2).

A large proportion of the explained variance is associated with factor F1 which accounts for 19.34% of the variance. Factor F1 includes seven items that describe the frequency, appropriateness, promptness, and adequacy of feedback received by students. For example, feedback is given promptly after an assessment (Item 19). Factor F2 explains 14.12% of the variance and includes five items describing the assessment environment that relate to students’ learning and performance. For example, the assessment system supports my learning (Item 36). Factor F3 explains 13.19% of the variance and comprises five items regarding information on assessment. For example, students receive clear information about the assessment (Item 15). Factor F4 explains 10.07% of the variance and contains three items that describe the existing assessment system/procedure. For example, students are adequately assessed (Item 2) (Table 2).

An overall Cronbach's *α* of 0.89 indicated that the AEQ is a reliable instrument with good internal consistency. With Cronbach's *α* ranging from 0.71 to 0.87 (Table 3), the four factors/subscales within the AEQ also have acceptable to high reliability or internal consistency (28, 31).

Item-to-total correlation was above 0.40 for 17 of the 20 items, with a mean of 0.50. This reflects good item discrimination.

The findings of mean total score for the entire AEQ as well as mean score for the individual AEQ items of approximately 67% indicated a moderately favourable assessment environment.

The subscale of ‘feedback mechanism’ recorded the lowest mean (2.39/4.00 or 59.75%) whereas ‘assessment system/procedure’ subscale scored the highest mean (2.92/4.00 or 73.00%). The low mean of the ‘feedback mechanism’ subscale could trigger an alarm for the faculty to re-examine the feedback mechanism within the assessment program. Meanwhile, the high mean of ‘the assessment system/procedure’ subscale could inform the faculty that students perceived the assessment system/procedure as favourable. Such findings suggested that the AEQ might be used as a screening tool to identify weaknesses and strengths related to the assessment environment of the undergraduate medical program.

Significant differences among the AEQ scores from students of different academic years indicated that students of different academic years had significantly different perceptions of the assessment environment. The mean AEQ score of Phase 2 students was significantly higher compared to students in Phase 1, Phase 3A, and Phase 3B. This finding showed Phase 2 students perceived the assessment environment more favourably compared to students of other academic years. Possible explanations included: 1) Phase 1 students were still trying to adjust to the new assessment environment of a medical school, and 2) clinical students in Phase 3A and Phase 3B moved from one setting to another in clinical rotations, depriving learners of extended teacher-learner relationships and creating barriers to effective feedback. However, the reason(s) for this difference was unclear and requires further data analysis, which is beyond the scope of this paper. No significant difference between the AEQ scores of male and female students from independent sample *t*-tests implied sex had no influence on students’ perceptions of the assessment environment. According to Messick (32), differences in means across groups supported an instrument's construct validity.

### Limitations of the study

The authors acknowledge several limitations of this study. Although the study population was drawn from medical students of different academic years, pre-university background, and sex, and the sample size of *n=*611 is adequate for statistical analysis, the instrument was only used to collect data from a single institution. Hence, generalisability of the findings from this study is limited. There may also be limitations in applying the new instrument to other medical institutions, which may vary in terms of medical curriculum, learning culture, delivery of instruction, and assessment program. With regards to validity, face and content validity were addressed and construct validity in terms of factor structure of the AEQ was examined. Other sources of validity evidence need to be explored to enhance the validity of the new instrument. On reliability of the instrument, only internal consistency was examined. Due to the busy schedule of the medical students, reliability over time in terms of test-retest reliability was not addressed.

### Recommendations for future research

To further validate the new instrument, it is suggested that the AEQ be used to collect data from different medical schools across different cultures to test its robustness in terms of its construct validity and internal consistency. Further analysis of data collected in this study to compare mean scores of selected subscales of the AEQ across different demographic background such as academic years, sex, and pre-university background are also suggested. Collection of qualitative data via focus group interviews is suggested to supplement the quantitative data collected from the AEQ to gain further insights and understanding into medical students’ perception of the assessment environment.

## Conclusions

The AEQ appeared to be a valid and reliable instrument. As far as validity of the instrument was concerned, there was evidence of its face, content, and construct validity. As for reliability, the overall instrument, as well as its four subscales, showed good internal consistency reliability. Initial validation of the instrument supported its use to measure students’ perceptions of the assessment environment in an undergraduate medical program. Mean score for the AEQ gave an indication of the assessment environment, whereas mean score of each subscale suggested the AEQ could be used as a screening tool to identify strength or weakness within the assessment environment.

## Appendix 1

**Table:** The Assessment Environment Questionnaire (AEQ) For each statement below, please CIRCLE your level of agreement.

No	Statement	Strongly disagree [1]	Disagree [2]	Agree [3]	Strongly agree [4]
1	Assessment in the MBBS program is conducted fairly.	[1]	[2]	[3]	[4]
2	Students are adequately assessed.	[1]	[2]	[3]	[4]
3	There is sufficient time gap(s) between exam papers.	[1]	[2]	[3]	[4]
4	Learning outcomes are appropriately assessed.	[1]	[2]	[3]	[4]
5	Students’ practical skills are appropriately assessed using OSPE.	[1]	[2]	[3]	[4]
6	A description of how individual assessments and exams contribute to the total score is made known to students.	[1]	[2]	[3]	[4]
7	I received information about what is expected of me in any exam/assessment.	[1]	[2]	[3]	[4]
8	I received feedback on my performance for continuous assessment.	[1]	[2]	[3]	[4]
9	The assessment system encourages me to reflect on my own performance.	[1]	[2]	[3]	[4]
10	Feedback is given to students irrespective of performance.	[1]	[2]	[3]	[4]
11	There are too many assessments in the MBBS program.	[1]	[2]	[3]	[4]
12	Assessments are set at an appropriate level of difficulty.	[1]	[2]	[3]	[4]
13	I do NOT have enough time to reflect on my learning.	[1]	[2]	[3]	[4]
14	There is a range of assessment methods/tools being used to assess a wide variety of knowledge, skills and behaviour.	[1]	[2]	[3]	[4]
15	Students receive clear information about assessment.	[1]	[2]	[3]	[4]
16	I understand the assessment criteria for all the tests/exams I took.	[1]	[2]	[3]	[4]
17	I received feedback on my performance for final exams.	[1]	[2]	[3]	[4]
18	Feedback from assessors about my performance is adequate.	[1]	[2]	[3]	[4]
19	Feedback is given promptly after an assessment.	[1]	[2]	[3]	[4]
20	There is a balanced mix of continuous assessments and final exams.	[1]	[2]	[3]	[4]
21	Topics that have more learning outcomes are tested more extensively.	[1]	[2]	[3]	[4]
22	The assessment system gives me an idea on where I stand in comparison to my peers.	[1]	[2]	[3]	[4]
23	Assessment format chosen for each learning outcome is appropriate (e.g., MCQs for testing knowledge and OSPE/OSCE for testing skills).	[1]	[2]	[3]	[4]
24	Students’ professional behaviour is appropriately assessed in CFCS debriefings.	[1]	[2]	[3]	[4]
25	Assessment criteria are clearly defined.	[1]	[2]	[3]	[4]
26	The pass/fail border is clearly defined and made known to students.	[1]	[2]	[3]	[4]
27	The form of feedback I received matches the purposes of the assessment.	[1]	[2]	[3]	[4]
28	Feedback from assessors about my performance is appropriate.	[1]	[2]	[3]	[4]
29	I receive on-going feedback on my progress.	[1]	[2]	[3]	[4]
30	The pass/fail border is fair.	[1]	[2]	[3]	[4]
31	Learning outcomes are assessed at appropriate points during the curriculum.	[1]	[2]	[3]	[4]
32	Students’ knowledge is appropriately assessed using MCQs, SAQs and Essays format questions.	[1]	[2]	[3]	[4]
33	Students are given oral briefings prior to an exam.	[1]	[2]	[3]	[4]
34	I receive feedback on my work from a range of sources (e.g., teachers, peers).	[1]	[2]	[3]	[4]
35	The feedback I received helped me to improve my learning.	[1]	[2]	[3]	[4]
36	The assessment system supports my learning.	[1]	[2]	[3]	[4]
37	I know the requirements for me to progress from one phase to another.	[1]	[2]	[3]	[4]
38	Students’ clinical skills are appropriately assessed using OSCE, long case, short case.	[1]	[2]	[3]	[4]
39	I know the requirements for me to graduate.	[1]	[2]	[3]	[4]
40	The feedback I received helped me to improve my grades.	[1]	[2]	[3]	[4]

## Appendix 2

**Table:** Rotated component matrix^a^

		Factor/component					
		
Item No.	Statement	1	2	3	4	5	6
1	Assessment in the MBBS program is conducted fairly.	0.022	−0.038	0.699	0.066	0.122	0.042
2	Students are adequately assessed.	0.178	0.096	0.743	0.148	0.061	−0.041
3	There is sufficient time gap(s) between exam papers.	0.016	0.004	0.391	0.117	−0.214	0.475
4	Learning outcomes are appropriately assessed.	0.117	0.160	0.688	0.174	−0.001	0.030
5	Students’ practical skills are appropriately assessed using OSPE.	0.187	0.255	0.503	0.078	0.196	0.029
6	A description of how individual assessments and exams contribute to the total score is made known to students.	0.144	0.039	0.154	0.597	0.049	−0.168
7	I received information about what is expected of me in any exam/assessment.	0.216	0.069	0.199	0.522	0.157	0.145
8	I received feedback on my performance for continuous assessment.	0.565	0.208	0.184	0.288	0.027	−0.139
9	The assessment system encourages me to reflect on my own performance.	0.246	0.536	0.117	0.384	0.047	0.020
10	Feedback is given to students irrespective of performance.	0.432	0.196	0.158	0.369	−0.019	−0.260
11	There are too many assessments in the MBBS program.	0.290	−0.164	0.058	0.005	−0.171	−0.538
12	Assessments are set at an appropriate level of difficulty.	−0.011	0.246	0.219	0.198	−0.033	0.083
13	I do NOT have enough time to reflect on my learning.	0.028	−0.104	−0.061	−0.129	−0.044	−0.728
14	There is a range of assessment methods/tools being used to assess a wide variety of knowledge, skills and behaviour.	0.296	0.313	0.169	0.310	−0.003	−0.017
15	Students receive clear information about assessment.	0.246	0.213	0.090	0.677	0.058	0.183
16	I understand the assessment criteria for all the tests/exams I took.	0.178	0.158	−0.030	0.682	0.125	0.219
17	I received feedback on my performance for final exams.	0.734	0.077	0.098	0.172	0.024	−0.071
18	Feedback from assessors about my performance is adequate.	0.764	0.048	0.072	0.214	0.065	−0.040
19	Feedback is given promptly after an assessment.	0.781	0.011	0.027	0.118	0.078	−0.127
20	There is a balanced mix of continuous assessments and final exams.	0.410	−0.144	0.306	0.108	0.352	0.198
21	Topics that have more learning outcomes are tested more extensively.	0.236	0.190	0.393	−0.064	0.386	0.179
22	The assessment system gives me an idea on where I stand in comparison to my peers.	0.175	0.378	−0.013	0.251	0.149	−0.058
23	Assessment format chosen for each learning outcome is appropriate (e.g., MCQs for testing knowledge and OSPE/OSCE for testing skills).	0.224	0.104	0.337	0.109	0.393	0.275
24	Students’ professional behaviour is appropriately assessed in CFCS debriefings.	0.416	0.234	0.144	0.245	−0.002	0.005
25	Assessment criteria are clearly defined.	0.264	0.178	0.121	0.581	0.202	0.071
26	The pass/fail border is clearly defined and made known to students.	0.059	0.155	0.009	0.307	0.530	−0.099
27	The form of feedback I received matches the purposes of the assessment.	0.577	0.301	0.179	0.114	0.129	0.039
28	Feedback from assessors about my performance is appropriate.	0.664	0.197	0.036	0.080	0.277	0.039
29	I receive on-going feedback on my progress.	0.728	0.132	0.056	0.078	0.105	−0.029
30	The pass/fail border is fair.	0.111	0.024	0.080	0.248	0.553	0.125
31	Learning outcomes are assessed at appropriate points during the curriculum.	0.286	0.037	0.285	0.080	0.441	0.143
32	Students’ knowledge is appropriately assessed using MCQs, SAQs and Essays format questions.	0.138	0.243	0.480	−0.027	0.459	0.029
33	Students are given oral briefings prior to an exam.	−0.095	0.317	0.168	0.250	0.193	0.053
34	I receive feedback on my work from a range of sources (e.g., teachers, peers).	0.376	0.512	0.056	0.042	0.025	0.149
35	The feedback I received helped me to improve my learning.	0.318	0.712	0.076	0.007	0.064	0.135
36	The assessment system supports my learning.	0.121	0.629	0.191	0.221	0.139	0.082
37	I know the requirements for me to progress from one phase to another.	0.091	0.439	0.039	0.342	0.331	−0.012
38	Students’ clinical skills are appropriately assessed using OSCE, long case, short case.	−0.015	0.304	0.352	0.118	0.409	−0.174
39	I know the requirements for me to graduate.	0.014	0.157	−0.011	−0.067	0.623	−0.043
40	The feedback I received helped me to improve my grades.	0.350	0.630	0.114	−0.120	0.180	0.154
	Extraction method: Principal component analysis.						
	Rotation method: Varimax with Kaiser normalisation.^a^						

a Rotation converged in eight iterations.
